# CYP2J2 and Its Metabolites EETs Attenuate Insulin Resistance via Regulating Macrophage Polarization in Adipose Tissue

**DOI:** 10.1038/srep46743

**Published:** 2017-04-25

**Authors:** Meiyan Dai, Lujin Wu, Peihua Wang, Zheng Wen, Xizhen Xu, Dao Wen Wang

**Affiliations:** 1 Division of Cardiology, Department of Internal Medicine and Institute of Hypertension, Tongji Hospital, Tongji Medical College, Huazhong University of Science and Technology, Wuhan, China

## Abstract

Macrophages in adipose tissue are associated with obesity-induced low-grade inflammation, which contributed to insulin resistance and the related metabolic diseases. Previous studies demonstrated the beneficial effects of epoxyeicosatrienoic acids (EETs) on metabolic disorders and inflammation. Here we investigated the effects of CYP2J2-EETs-sEH metabolic pathway on insulin resistance in mice and the potential mechanisms. High fat diet (HFD)-induced obesity caused metabolic dysfunction with more weight gain, elevated glucose and lipids levels, impaired glucose tolerance and insulin sensitivity, while increase in EETs level by rAAV-mediated CYP2J2 overexpression, administration of sEH inhibit TUPS or EETs infusion significantly attenuated these metabolic disorders. EETs inhibited macrophages recruitment to adipose tissue and their switch to classically activated macrophage (M1) phenotype, while preserved the alternatively activated macrophage (M2) phenotype, which was accompanied by substantially reduced adipose tissue and systemic inflammation and insulin resistance. *In vitro* studies further clarified the effects of EETs on macrophage infiltration and polarization, and microarray assays showed that cAMP-EPAC signaling pathway was involved in these processes. Collectively, these results described key beneficial immune-regulatory properties and metabolic regulation of CYP2J2-EETs-sEH metabolic pathway, and indicated therapeutic potential of EETs in obesity-induced insulin resistance and related inflammatory diseases through modulating macrophage polarization targeting cAMP-EPAC signaling pathway.

Obesity is a prevalent metabolic disease characterized by excess accumulation of white adipose tissue (WAT) due to increased food intake combined with a sedentary lifestyle changes^1^. According to the World Health Organization (WHO), 39% of adults over 18 years of age are overweight and 13% are clinically obese, and more than 50 million obese children under the age of 5^2^. The pathophysiological impact of obesity far exceeds its cosmetic effect on body shape, the incidence of obesity related diseases such as type 2 diabetes, cardiovascular complications and cancer significantly increased, its impact on public health cannot be dismissed^3^. Adipose tissue macrophages (ATMs) associated low-grade inflammation in adipose tissue that promotes insulin resistance (IR), is increasingly appreciated as a major abnormality involved in diet-induced obesity^4^. The immune phenotype of ATMs in obesity has been demonstrated to shift from a more alternatively activated (M2) to a classically activated (M1) phenotype. M1 ATMs produce pro-inflammatory cytokines, such as tumor necrosis factor α (TNFα), interleukin (IL)-6, and monocyte chemoattractant protein (MCP)-1, thus contributing to the induction of insulin resistance. On the other hand, M2 ATMs, which are the major resident macrophages in lean adipose tissue, are reported to have a different gene expression profile, characterized by the relatively high expression of CD206, arginase-1, Mgl-I, and IL-10, which are involved in the repair or remodeling of tissues^5^. The consequences of this imbalance toward M1 recruitment into adipose tissue in obesity include insulin resistance and metabolic dysfunction. In line with these observations, several studies that target ATMs to attenuate the secretion of pro-inflammatory cytokines have shown beneficial effects with respect to insulin resistance. Thus, identification of key molecular mechanisms underlying local inflammation in obesity is of significant interest and may lead to effective strategies to treat obesity and insulin resistance.

CYP2J2 is a cytochrome P450 epoxygenase that is widely expressed in various tissues and organs in human^6^. CYP2J2 metabolizes arachidonic acid (AA) to four biologically active epoxyeicosatrienoic acids (EETs): 5,6-, 8,9-, 11,12-, and 14,15-EETs, which can be rapidly hydrolyzed to biologically less active dihydroxyeicosatrienoic acids (DHETs) by soluble epoxide hydrolase (sEH). Inhibitors of sEH prevent the conversion of EETs to DHETs, resulting in stabilized EETs levels^7^. Recent studies demonstrated that EETs played important roles in metabolic syndrome and diabetes^8^,^9^,^10^,^11^,^12^. CYP2J2 gene delivery attenuated metabolic dysfunction by reducing hepatic inflammation^9^,^13^, improved STZ-induced cardiac cardiomyopathy^14^, attenuated vascular dysfunction and adiposity^10^, modulated liver inflammation and autophagy in obesity^9^,^11^. Thus, EETs can inhibit cardiovascular, renal and liver diseases. However, little is known about how CYP2J2-EETs-sEH system orchestrates the adipose tissue function and metabolism, and the mechanisms by which EETs attenuate insulin resistance need to be further explored. Here we hypothesize that EETs regulate adipose tissue macrophages polarization and prevent HFD-induced insulin resistance in mice. To test the hypothesis, the increase in EETs levels by overexpression of CYP2J2 or administration of sEH inhibitor TUPS, or directly by infusion of EETs with osmic minipump in mice, was induced to explore the effects of EETs on adipose tissue macrophages polarization.

## Research Design and Methods

### Construction and Preparation of Recombinant Adeno-associated Virus (rAAV)

rAAV-CYP2J2 and rAAV-GFP were prepared by triple plasmids co-transfection in HEK293 cells as previously described^15^.

### Animals

All animal care and experimental procedures were approved by the Experimental Animal Research Committee of Tongji Medical College, Huazhong University of Science & Technology, and in strict accordance with the recommendations in the Guide for the Care and Use of Laboratory Animals of the NIH.

Animal experiment 1: four weeks old C57BL/6 male mice were randomly assigned to either a high fat diet (HFD) (D12492, 60% energy by fat, Beijing HFK Bio-Technology, China) or normal diet (ND) (D12450B, 10% energy by fat, Beijing HFK Bio-Technology, China) for 16 weeks. D12450B 10 kcal % low-fat feed used as the control for D12492 60 kcal % high-fat feed. Both have the same recipe ingredients and total calories (kcal) content. 120 mice were then randomly assigned to 8 groups (n = 15): ND, ND + rAAV-GFP, ND + rAAV-CYP2J2, ND + TUPS, HFD, HFD + rAAV-GFP, HFD + rAAV-CYP2J2 and HFD + TUPS. Animals received injection of rAAV-CYP2J2 and rAAV-GFP (1 × 10^11^ p.f.u) through tail vein of 4 week old mice, and TUPS (an inhibitor of sEH) was administered in drinking water (10 mg/L) from the beginning to the end of the experiment.

Animal experiment 2: four weeks old C57BL/6 male mice were randomly assigned to either a high fat diet (HFD) (60% fat) or normal diet (ND) (10% fat) for 16 weeks. Animals were divided into experimental groups (n = 15 per group) as follows: ND, HFD, HFD + 11,12-EET and HFD + 14,15-EET. 11,12-EET and 14,15-EET were given at a rate of 15 μg/kg/day 12 weeks after HFD by an osmotic mini-pump as previously described^16^. Body weight, food and water intake were monitored every two weeks.

### Glucose and Insulin Tolerance Tests

After 16 weeks of HFD, glucose tolerance test (GTT) and insulin tolerance test (ITT) measurements were performed as previously described^17^.

### Cell Cultures

Bone marrow-derived macrophages (BMDMs) were isolated from femurs and tibias of C57BL/6 mice, and cultured as previously described^18^. 3T3-L1 pre-adipocytes obtained from ACTT were cultured and differentiated into adipocyte as previously described^19^.

In the indirect co-culture experiments, 3T3-L1 pre-adipocytes were differentiated in 6-well plates and then treated with conditioned medium from macrophages. Cells were cultured overnight before insulin signaling study. FFA/BSA complex solution was prepared as previously described^20^.

### Migration Assay

Chemotaxis assay of BMDMs was performed using Boyden chambers as described previously^21^. The BMDMs were placed in the upper chamber, whereas adipocytes conditioned medium (CM) was placed in the lower chamber. After incubation for 3 h, transmigrated cells were fixed in formalin and stained with crystal violet, and counted under microscope.

### Measurement of Metabolic Parameters

Serum levels of glucose, TG and TC were measured using commercially available kits from Bioassays Systems. Serum insulin level was determined using Ultra-Sensitive Mouse Insulin ELISA kit. IL-1β, IL-6, MCP-1 and TNF-α levels in serum were measured as manufacturer’s instructions.

### Evaluation of Serum and Urine EETs and DHETs

Serum and urine samples from all mice were collected. ELISA kits (Detroit R&D, Detroit, MI) were used to determine concentrations of 11,12-EET and its stable metabolite11,12-DHETs as manufacturer’s instructions.

### Isolation of Stromal Vascular Fraction and Flow Cytometry Analyses

The stromal vascular fraction (SVF) from murine adipose tissue was isolated as previously described^22^. Mouse SVF were hybridized with CD3-FITC, CD4-APC, and CD8-PE antibodies or F4/80-PE/Cy5, CD11b-FITC, CD206-PE and CD11c-PE antibodies and analyzed on a BD LSRFortessa using FACSDiva Software to detect CD3^+^CD4^+^CD8^−^ and CD3^+^CD4^−^CD8^+^ T cells and F4/80^+^CD11b^+^CD206^+^, F4/80^+^CD11b^+^CD11c^+^ ATMs, using appropriate compensation controls to set gates.

### Blood Leukocytes

Leukocyte subsets were identified from whole blood as previously described^23^. Monocytes were identified as F4/80^+^CD11b^+^ and subsets as Ly6G^+^ and Ly6G^−^, neutrophils were identified as F4/80^−^CD11b^+^Ly6G^+^.

### Histological Analysis

Hematoxylin/eosin (HE) staining, CD68 and CYP2J2 immunohistochemistry were performed in paraffin-embedded epididymal adipose tissue (eAT). Adipose tissue immunofluorescence staining was performed as previously described^24^. Images were taken with a digital camera (Nikon DS-2Mv). The analysis was performed by Image Pro Plus software.

### Protein Extraction and Western Blotting

For assessing insulin-induced Akt phosphorylation (Ser473) in liver, epididymal fat tissue, mice were injected with insulin (1 U/kg i.p.). Then, 5 min after the insulin injection, liver and epididymal fat tissues were harvested and lysed in assay buffer^17^. Western blot were performed using various antibodies. Bands were quantified by densitometry using Quantity One software (Bio-Rad, Hercules, CA).

### RNA Extraction, cDNA Synthesis, and Quantitative PCR

Total RNA was extracted using Trizol reagent (Invitrogen, USA) and reverse transcribed as described previously^20^. Quantitative real-time PCR was performed on the Applied Biosystems 7900 system. The sequences of primers used were showed in Supplemental Table 1. GAPDH served as internal normalization control.

### Microarray

RNA for analysis of expression by microarray was isolated from BMDMs. The sample labeling, hybridization, staining and scanning procedures were carried out at Shanghai Biotechnology Corporation using Agilent Whole Mouse Genome Oligo Microarrays (4 × 44 k). The experimental data were analyzed using GeneSpring Software GX 12.6.1 (Agilent technologies, Santa Clara, CA, US) and normalized by the Quantile method. The 2-fold change between two groups was the threshold for significant regulation.

### Statistical Analysis

The results obtained were expressed as the mean ± S.E.M. *P* values were calculated with a two-tailed unpaired Student’s *t*-test or, for the comparison of more than two conditions, with a one- or two-way ANOVA followed by Bonferroni’s or Tukey’s post-test. *P* < 0.05 was considered significant.

## Results

### Gene Delivery of CYP2J2 and sEH Inhibitor TUPS Increased Circulating EETs Level in Mice

**Figure 1:**
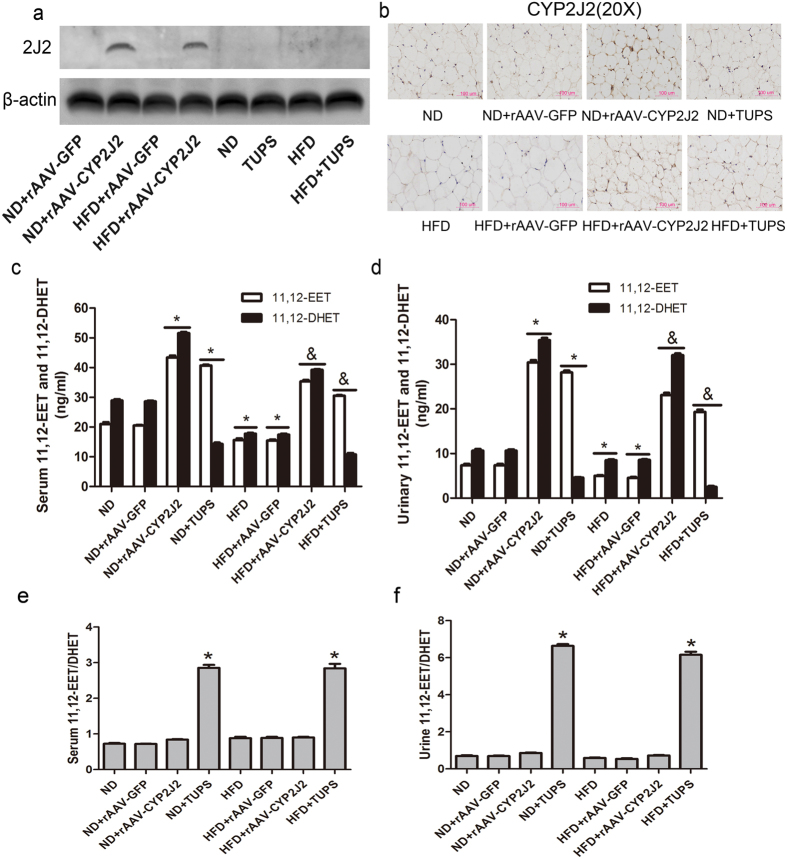
Gene delivery of CYP2J2 and sEH inhibitor TUPS increased circulating EETs level in mice. (**a**) CYP2J2 was abundance in adipose tissue after rAAV-CYP2J2 injection. (**b**) Representative images of CYP2J2 staining in adipose tissue. The concentration of 11,12-EET and the corresponding 11,12-DHET in serum (**c**) and urine (**d**) of mice on ND or HFD, and treated with rAAV-CYP2J2 and TUPS. 11,12 EET/DHET was calculated as index of sEH activity (**e,f**). (n = 15 for each group; *p < 0.05 *vs* ND or ND+ rAAV-GFP; ^&^*p* < 0.05 *vs* ND+ rAAV-CYP2J2 or ND+ TUPS).

16 weeks after rAAV-CYP2J2 injection, CYP2J2 expression in adipose tissue was abundant as evaluated by western blot (Fig. 1a). Immunohistochemical results further confirmed this trendency in CYP2J2 expression in adipose tissue in CYP2J2 gene delivery group but not in other mice (Fig. 1b). We further determined levels of 11,12-EETs and their corresponding 11,12-DHETs in serum and urine, respectively. As Fig. 1c,d depicted, rAAV-CYP2J2 injection and TUPS administration caused a significant elevation in both serum and urine level of 11,12-EET, the corresponding 11,12-DHET level was elevated in mice injected with rAAV-CYP2J2 while declined in TUPS treated mice. Interestingly, we also found that 11,12-EET level in HFD-induced mice was lower than that in mice treated with ND. 11,12-EET/DHET in serum and urine were calculated as the sEH activity (Figure e,f), results showed that sEH inhibitor obviously inhibit sEH activity, while HFD had no effect. These results demonstrated that overexpression of CYP2J2 and inhibition of sEH activity by TUPS increased EETs level *in vivo*.

### CYP2J2 and TUPS Administration Attenuated Metabolic Dysfunction in HFD Mice

**Figure 2:**
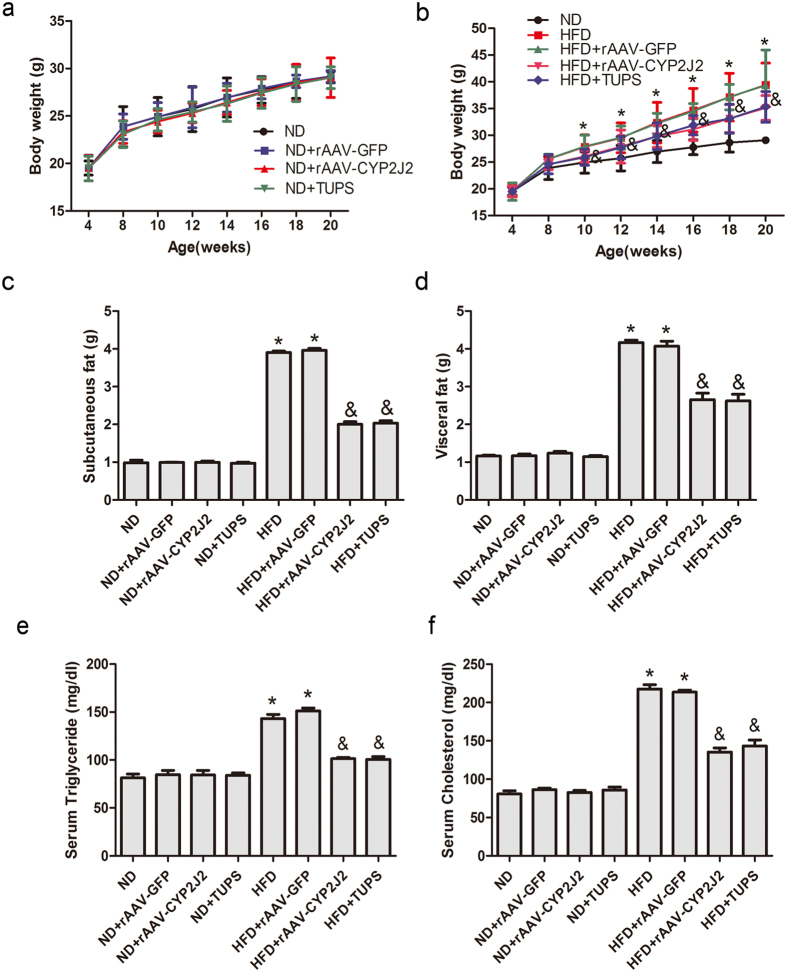
CYP2J2 and TUPS administration attenuated metabolic dysfunction in HFD mice. (**a,b**) The curves of body weight gain over time. The subcutaneous (**c**) and visceral (**d**) fat content of mice under various treatment conditions. The concentration of serum triglyceride (**e**) and cholesterol (**f**) in mice. (n = 15 for each group; *p < 0.05 *vs* ND; ^&^*p* < 0.05 *vs* HFD).

We first analyzed metabolic parameters in mice. Faster body weight gain was observed over time in mice fed with HFD, which was obviously from 10 weeks following a diet onwards. CYP2J2 and TUPS administration attenuated these effects (Fig. 2a,b), and interestingly, decreased subcutaneous and visceral fat mass were seen in HFD mice (Fig. 2c,d). However, food consumption and water intake were similar among these groups (Supplemental Fig. 1a,b), which suggest that the difference in body weight is not caused by food intake. We conducted real-time PCR experiment to explore the effect of EETs on the gene expression of energy expenditure. Results showed that HFD decreased the expression of UCP-1, UCP-2 and PGC-1α in adipose tissue, EETs attenuated these effects (Supplemental Fig. 1c), which indicated that EETs decreased the body weight partially via increasing energy utilization. Mice with HFD developed hyperlipidemia, as shown with increased serum triglycerides and cholesterol levels (Fig. 2e,f), while rAAV-CYP2J2 and TUPS administration normalized the changes in blood lipids. Consistently, infusion of EETs with mini-pump attenuated HFD-induced body weight gain and subcutaneous and visceral fat distribution, as well as the changes in levels of triglycerides and cholesterol, though some of them without significant difference (Supplemental Fig. 2). These data demonstrated that exogenous and endogenous EETs prevented the metabolic dysfunction in HFD mice.

### rAAV-CYP2J2 Treatment and TUPS Administration Attenuated HFD-induced Insulin Resistance

**Figure 3:**
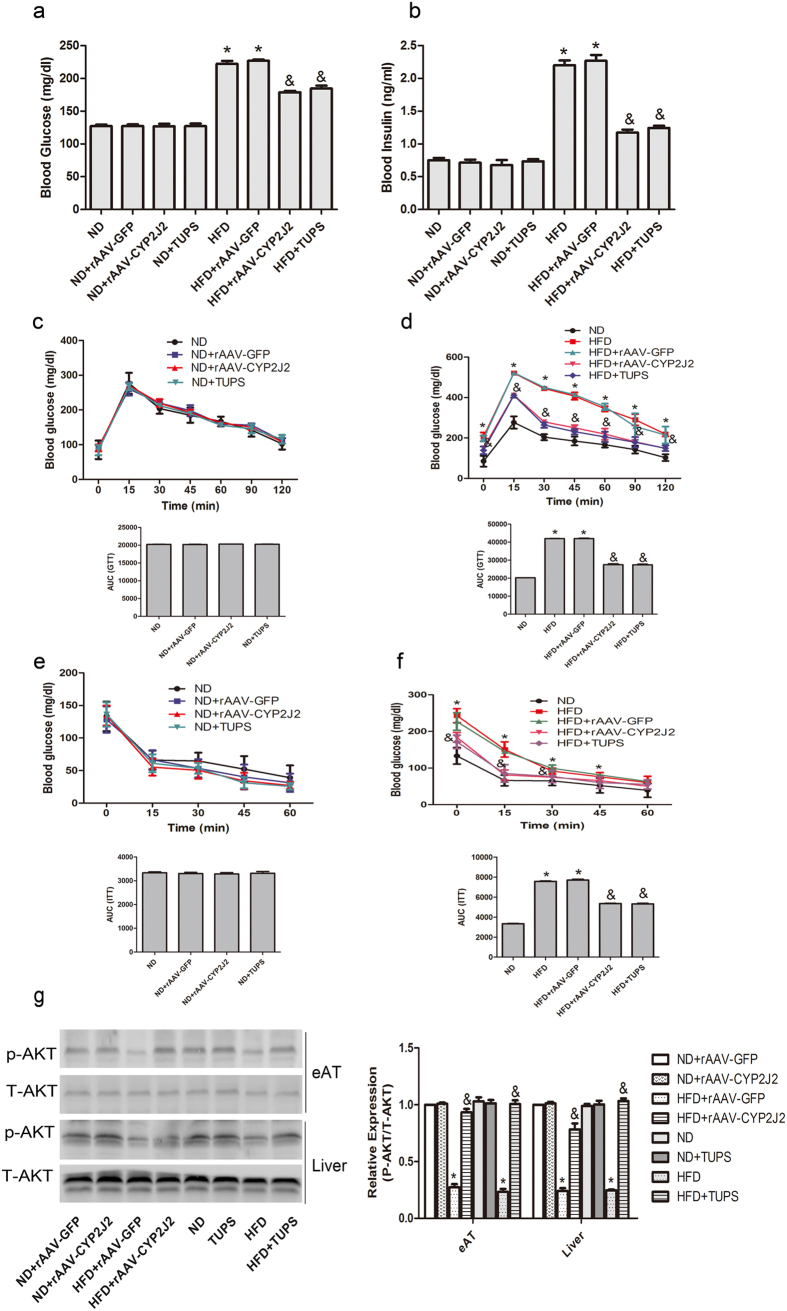
CYP2J2 treatment and TUPS administration attenuated HFD-induced insulin resistance. Serum concentration of glucose (**a**) and insulin (**b**) in mice after 16 weeks of ND or HFD. (**c–f**) GTT and ITT assay were performed in mice fed ND or HFD for 16 weeks. (**g**) Representative immunoblots and quantitation for Akt phosphorylation level in mice epididymal adipose tissue (eAT) and liver. (n = 15 for each group; *p < 0.05 *vs* ND or ND+ rAAV-GFP; ^&^*p* < 0.05 *vs* HFD or HFD+ rAAV-GFP).

We next examined the roles of EETs in regulating glucose and insulin homeostasis. After 16 weeks of HFD, fasting glucose and insulin levels were substantially increased, suggesting a reduction in insulin sensitivity; rAAV-CYP2J2 treatment and TUPS administration attenuated these disorders (Fig. 3a,b). Furthermore, glucose tolerance test (GTT) and insulin tolerance test (ITT) further verified the pivotal role of CYP2J2 and TUPS in regulating insulin sensitivity when compared with HFD alone (Fig. 3c–f). Moreover, insulin-induced Akt phosphorylation in epididymal fat and liver were decreased in HFD, while rAAV-CYP2J2 injection and TUPS administration improved it (Fig. 3g). In accordance, infusion of EETs with mini-pump attenuated HFD induced insulin resistance characterized by decreased plasma glucose and insulin levels, improved insulin sensitivity by GTT and ITT, and preserved insulin-induced Akt phosphorylation level (Supplemental Fig. 3). Together, these findings indicated that EETs prevented HFD-induced insulin resistance.

### rAAV-CYP2J2 and TUPS Attenuated HFD-induced Inflammation in Adipose Tissues

**Figure 4:**
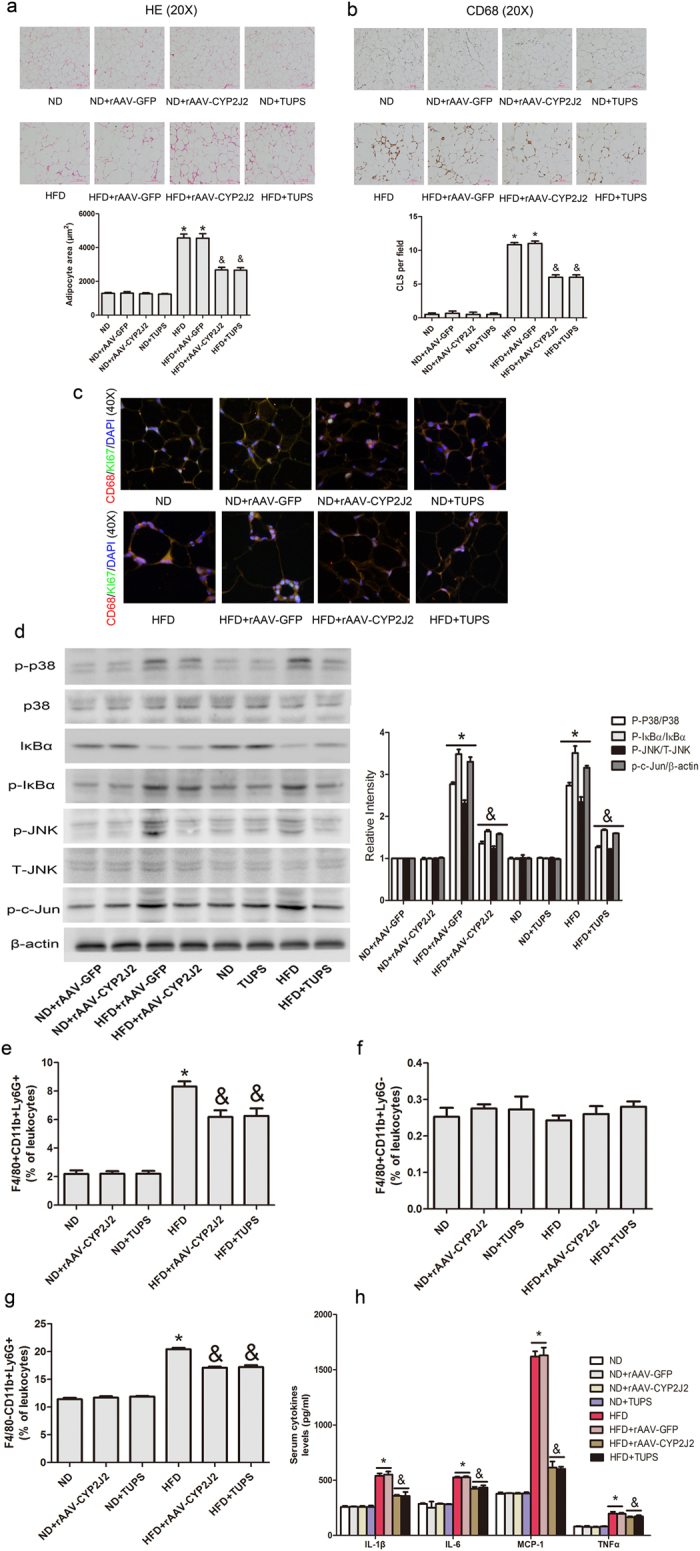
CYP2J2 and TUPS attenuated HFD-induced inflammation in adipose tissue. (**a**) Representative images of HE staining in adipose tissue and the calculated average adipocyte area. (**b**) Representative CD68 immunohistochemical staining and the numbers of CLS in adipose tissue with indicated interventions. (**c**) Representative images of macrophage proliferation staining with CD68 and Ki67. (**d**) Representative immunoblots and quantitation for MAPK and NF-κB signaling pathways in adipose tissue. Pro-inflammatory (**e**) and anti-inflammatory (**f**) monocytes and neutrophils (**g**) levels in the circulation. (**h**) ELISA analysis of inflammatory cytokines in serum. (n = 15 for each group; *p < 0.05 *vs* ND or ND+ rAAV-GFP; ^&^*p* < 0.05 *vs* HFD or HFD+ rAAV-GFP).

We further examined the extent of inflammation in adipose tissue and plasma levels. HE staining of adipose tissue showed increased adipocyte size and, moreover, crown-like structures (CLS), representing an accumulation of macrophages around dead adipocytes, were apparent in HFD mice; rAAV-CYP2J2 overexpression and TUPS administration, or EETs infusion significantly attenuated these effects (Fig. 4a,b and Supplemental Fig. 4a,b). To test whether ATM proliferation increases in the inflammatory setting of obesity, WAT were stained with an antibody against the proliferation marker Ki67, which is a protein expressed during all active phases of the cell cycle^23^. Interestingly, most of the Ki67 staining was observed in macrophages-rich region in WAT termed crown-like structures (CLSs). EETs inhibited the proliferation of macrophages in WAT (Fig. 4c and Supplemental Fig. 4c). In line with these data, rAAV-CYP2J2 overexpression and TUPS administration, or EETs infusion reduced HFD-induced adipose tissue MAPK and NF-κB signaling pathway (Fig. 4d and Supplemental Fig. 4d).

We analyzed the numbers of circulating F4/80^+^CD11b^+^Gr1^+^ (pro-inflammatory) and F4/80^+^CD11b^+^Gr1^−^ (anti-inflammatory) monocytes and F4/80^−^CD11b^+^Gr1^+^ neutrophils in ND and HFD mice. Because the disturbance of the GFP fluorescent, the rAAV-GFP groups were not included in the anslysis of FCM. Results showed that numbers of monocyte and neutrophils were comparable among ND mice, but under HFD conditions, pro-inflammatory monocytes and neutrophils were elevated, but rAAV-CYP2J2 treatment and TUPS administration significantly inhibited these effects. Meanwhile, rAAV-CYP2J2 and TUPS preserved the population of anti-inflammatory monocytes in the circulation (Fig. 4e–g). Systemic inflammation status was also detected, and results showed that rAAV-CYP2J2 and TUPS, or EETs infusion substantially reduced serum levels of these pro-inflammatory cytokines, IL-1β, IL-6, MCP-1 and TNFα (Fig. 4h and Supplemental Fig. 4e). Taken together, these data indicated that EETs inhibited HFD-induced inflammation.

### EETs and TUPS Administration Attenuated Chemokine-mediated Macrophage Infiltration and Improved Insulin Sensitivity

**Figure 5:**
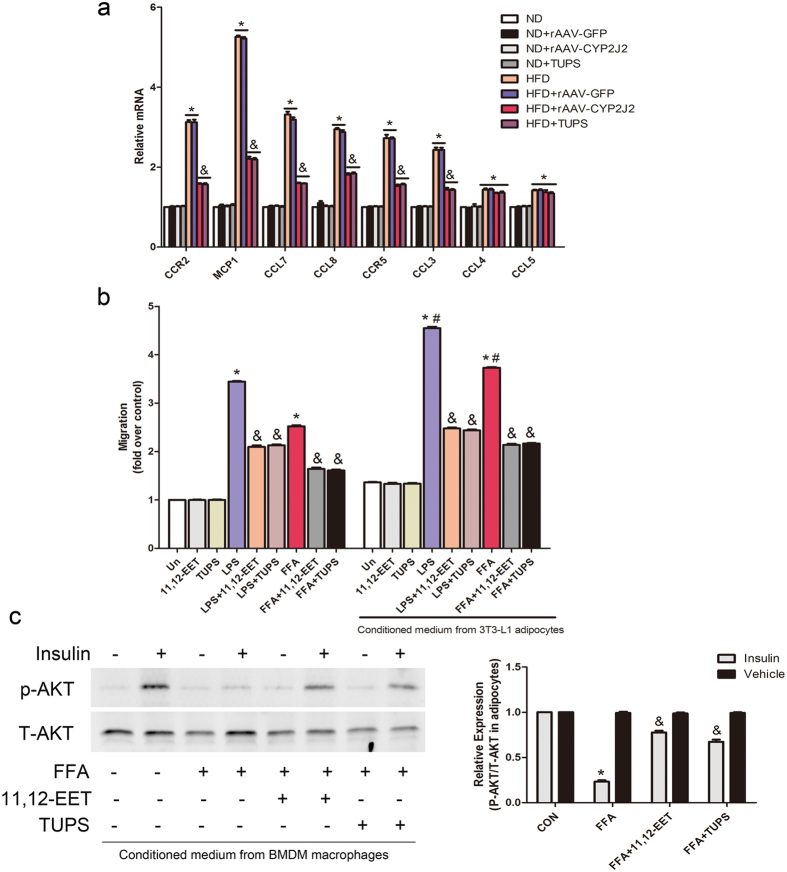
EETs and TUPS administration attenuated chemokine-mediated macrophage infiltration and improved insulin sensitivity in adipocyte. (**a**) Chemokines and chemokine receptors expression in adipose tissue were analyzed by quantitative real-time PCR. (**b**) BMDMs migration under indicted interventions. (**c**) Representative immunoblots and quantitation for Akt phosphorylation level in 3T3-L1 differentiated adipocytes incubated with conditioned medium from macrophages. (n = 15 for each group; *p < 0.05 *vs* ND; ^&^*P* < 0.05 *vs* HFD in (**a**) n = 3 for each group; *p < 0.05 *vs* control; ^&^*p* < 0.05 *vs* LPS or FFA in (**b** and **c**); ^#^p < 0.05 *vs* stimulus without condition medium in (**b**).

The chemokine signaling network is crucial in ATM functions^18^. We therefore analyzed the expression of several important chemokines and their receptors in adipose tissue. Gene expression of CCL2, CCL7, CCL8 and CCL3, as well as the chemokine receptors CCR2 and CCR5 were robustly increased in WAT of HFD mice, CYP2J2 overexpression and TUPS administration reduced these effects, while had little effect on CCL4 and CCL5 expression (Fig. 5a). Given the increase in chemokine and chemokine receptors *in vivo*, we next examined the effect of EETs on the migration of primary macrophage *in vitro*. BMDMs were induced with conditioned medium (CM) collected from fully differentiated 3T3-L1 adipocytes that treated with various stimuli. Results showed that LPS/FFA induced macrophages migration, which was exacerbated with CM from adipocytes, suggesting that activated adipocytes secreted chemokines that had potent effects to induce macrophages migration; 11,12-EET or TUPS inhibited these effects (Fig. 5b). 11,12-EET or TUPS prevented the impaired insulin-induced Akt phosphorylation in 3T3-L1 differentiated adipocytes treated with conditioned medium from macrophages (Fig. 5c). Taken together, these data indicate that EETs reduce chemokine expression that could mediate obesity-induced macrophage recruitment and activation in adipose tissue, and thus preserve the insulin sensitivity in adipocytes.

### CYP2J2 Overexpression and TUPS Administration Regulated Macrophages Polarization in Adipose Tissue

**Figure 6:**
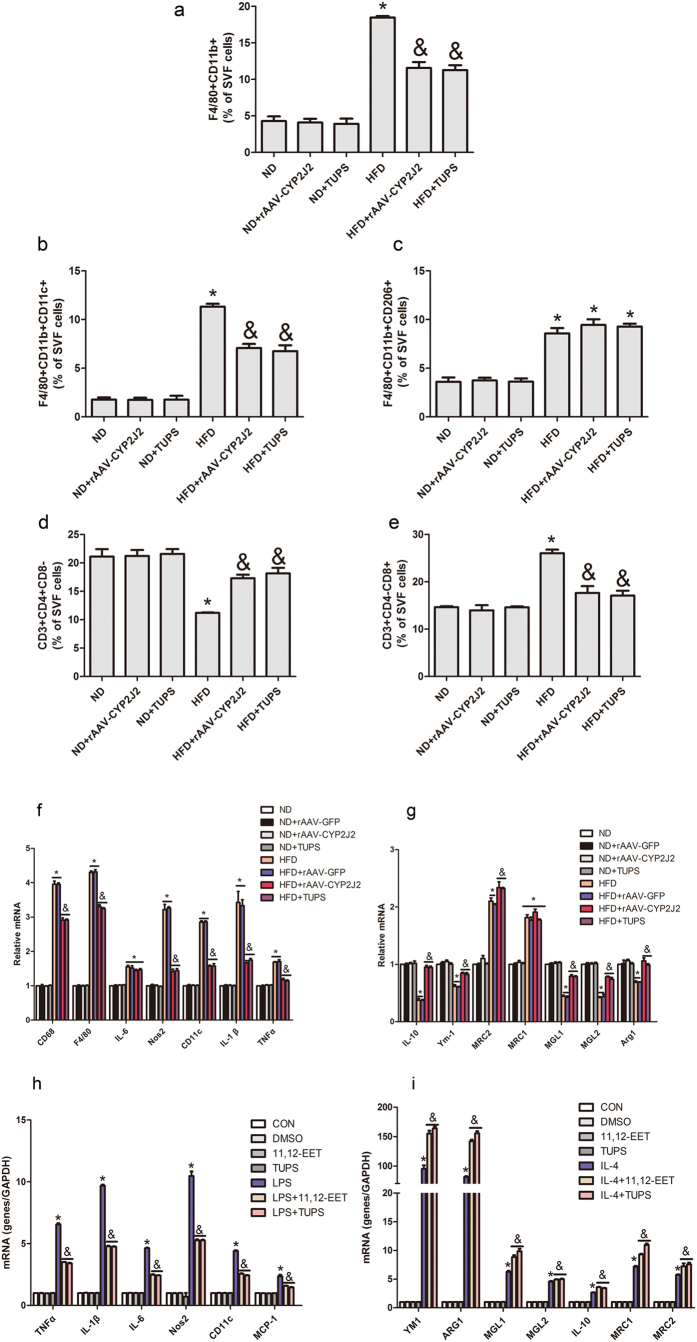
CYP2J2 overexpression and TUPS administration regulated macrophages polarization in adipose tissue. (**a–c**) Proportion of total macrophages and the respectively M1 and M2 phenotypes in SVF. (**d,e**) Proportion of CD4^+^ or CD8^+^ T cells in SVF. (**f–i**) Gene expression of M1 and M2 macrophages related molecules in adipose tissue and BMDMs were analyzed by quantitative real-time PCR. (n = 15 for each group; *p < 0.05 *vs* ND; ^&^*p* < 0.05 *vs* HFD in (**a–g**); *p < 0.05 *vs* control; ^&^*p* < 0.05 *vs* LPS or IL-4 in **(h,i**).

Adipose tissue macrophages accumulation and polarization are crucial to insulin resistance^25^,^26^. We next examined the phenotype of ATM in mice. The flow cytometric analyses of the SVF showed that the percentages of F4/80^+^CD11b^+^ cells were increased in HFD mice, while CYP2J2 overexpression and TUPS administration attenuated these effects (Fig. 6a). We used antibodies against CD11c and CD206 to discriminate M1 and M2 macrophages, respectively. The increased percentages of CD11c-positive M1 ATMs in HFD mice were reduced after rAAV-CYP2J2 and TUPS treatments, while in contrast the percentages of CD206-positive M2 ATMs were well preserved in mice with CYP2J2 and TUPS treatments (Fig. 6b,c). In addition, the percentages of CD8^+^ T cells were higher in mice with HFD treatment while CD4^+^ T cells were reduced; CYP2J2 overexpression and TUPS administration reversed this phenotype effectively (Fig. 6d,e). We subsequently validated the gene expression profiles in WAT. All the examined M1 markers (CD68, F4/80, IL-6, Nos2, CD11c, IL-1β and TNFα) were dramatically elevated under HFD conditions, whereas overexpression of rAAV-CYP2J2 and TUPS administration attenuated such elevations except IL-6 level (Fig. 6f). In contrast, the mRNA levels of M2 markers (IL-10, YM-1, MGL-1, MGL-2 and ARG-1) were well preserved by overexpression of rAAV-CYP2J2 and TUPS administration in HFD treated mice, with MRC-2 and MRC-1 further elevated upon HFD feeding (Fig. 6g). Finally, we sought to test the effects of EETs on macrophages polarization *in vitro*. Exogenous 11,12-EET and TUPS significantly inhibited LPS induced M1 polarization while promoted IL-4 induced M2 polarization (Fig. 6h,i). These results indicate that EETs regulated the balance of M1/M2 macrophages polarization and thereby maintained immune homeostasis in the adipose tissue.

### EETs Regulated Macrophages Polarization via Preventing cAMP-EPAC Signaling Pathway

**Figure 7:**
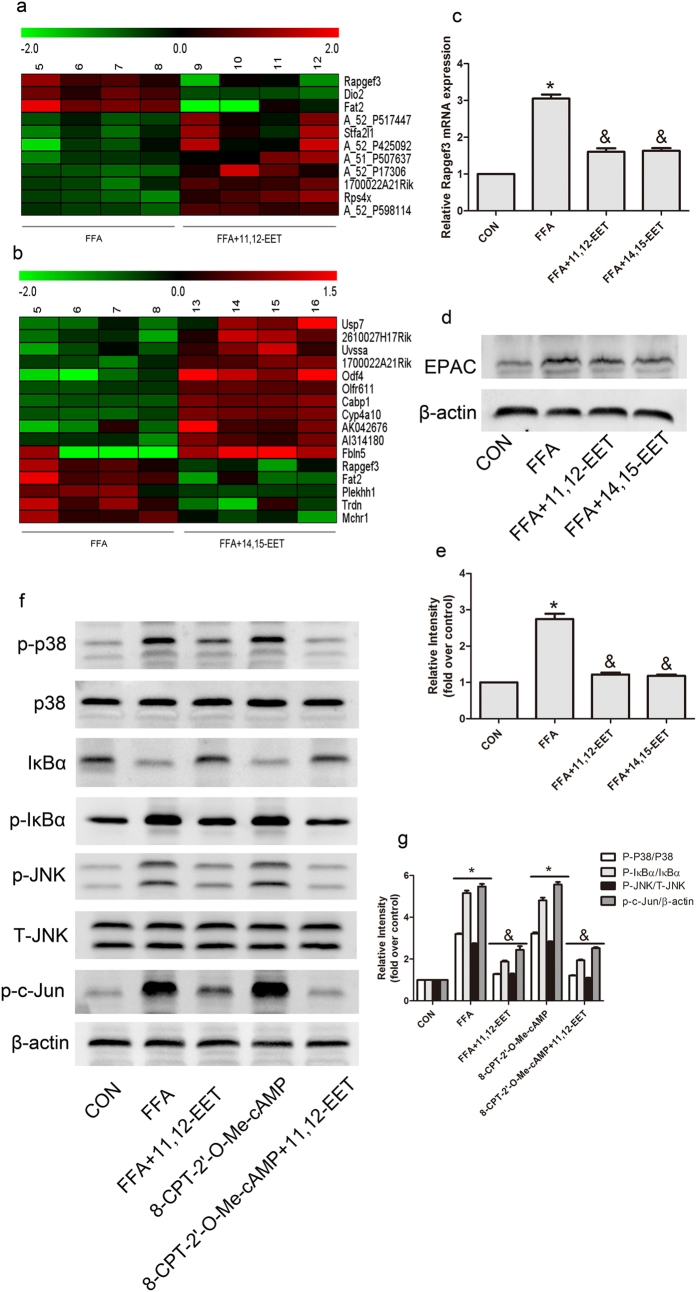
EETs regulated macrophages polarization via preventing cAMP-EPAC signaling pathway. (**a,b**) Microarray of gene expression in BMDMs with indicated interventions. Validation expression of Rapgef3 was performed by quantitative real-time PCR and western blot (**c–e**). (**f,g**) Representative immunoblots and quantitation for MAPK and NF-κB signaling pathways in BMDMs with indicated treatment. (n = 4 for each group; *p < 0.05 *vs* control; ^&^*p* < 0.05 *vs* FFA or 8-CPT-2′-*O*-Me-cAMP).

To further explore the mechanisms of EETs on macrophages polarization *in vitro*, we compared gene expression in BMDM stimulated with FFA or FFA plus EETs (11,12-EET or 14,15-EET). Among several differentially expressed genes identified, we focus on gene RAPGEF3 due to its important role in energy homeostasis. Rapgef3 was significantly down-regulated by EETs under the condition of FFA treatment (Fig. 7a,b). Validation was performed by real-time PCR and Western blots. Results showed that stimulation with FFA increased mRNA level of Rapgef3 and protein level of exchange proteins directly activated by cAMP (EPAC) in BMDMs, and this response was blunted with EETs (Fig. 7c,e). Using EPAC specific cAMP agonist (8-CPT-2′-*O*-Me-cAMP) revealed that increased the level of EPAC by 8-CPT-2′-*O*-Me-cAMP mimicked the effect of FFA on the regulation of MAPK and NF-κB signaling pathway, EETs inhibited the expression of EPAC, thus attenuated the downstream inflammatory signaling pathway (Fig. 7f,g). These data suggest that cAMP-EPAC signaling pathway may play an important role in regulation of macrophage polarization and inflammation.

## Discussion

**Figure 8:**
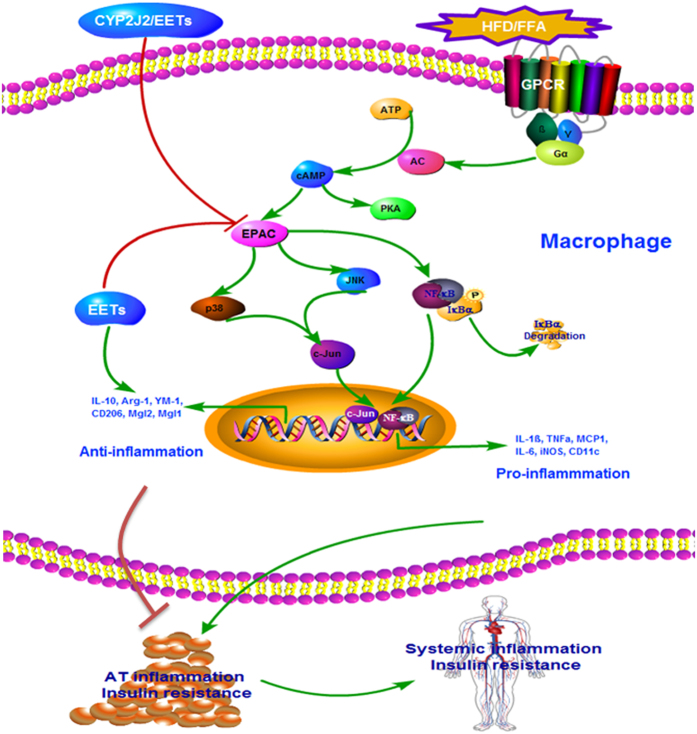
Schematic of mechanisms on CYP2J2/EETs mediated HFD/FFA induced inflammation and insulin resistance. HFD/FFA induced the expression of cAMP-EPAC and the downstream MAPK and NF-κB signaling pathways, which towards the adipose tissue macrophages to M1 pro-inflammatory phenotype and contributed to the systemic inflammation and insulin resistance. Exogenous and endogenous EETs inhibited macrophages accumulation and shifted the macrophages phenotype from M1 to M2 via preventing the expression of EPAC in the metabolic environment, which substantially attenuated adipose tissue and systemic inflammation and insulin resistance. (This figure was drawn by author Meiyan Dai using software of Pathway Builder Tool 2.0).

The present study investigated the effects of CYP2J2-EETs-sEH metabolic pathway at the interface between the metabolic and immunological arenas. HFD for 16 weeks in mice induced profound insulin resistance and diet-induced obesity associated chronic inflammatory response involving immunity changes^24^. Increased EETs levels in mice via rAAV-CYP2J2 gene therapy, TUPS or direct chronic infusion attenuated HFD induced metabolic dysfunction, as shown with diminished body weight gain, lower plasma glucose and lipids levels, reduced adipose tissue and systemic inflammation, as well as improved glucose tolerance and insulin sensitivity. The mechanisms of which were associated with regulating the balance of M1/M2 macrophage polarization in adipose tissue. Moreover, *in vitro* experiments further confirmed the effects of exogenous EETs on FFA-induced macrophages polarization and the potential mechanism was associated with cAMP-EPAC signaling pathway (Fig. 8).

Our and others studies revealed the protection effects of CYP2J2-EETs-sEH in metabolic homeostasis via targeting insulin reaction organs, such as liver^9^,^27^, pancreas^28^,^29^,^30^, heart^14^,^20^,^31^ and adipose tissue^11^,^32^. Pharmacological inhibition of epoxide hydrolase or deletion of the gene encoding soluble epoxide hydrolase (Ephx2) preserves islet cells in rodent models of type1 diabetes and enhances insulin sensitivity in models of type2 diabetes, as does administration of epoxyeicosatrienoic acids or their stable analogues. In humans, circulating concentrations of epoxyeicosatrienoic acids correlate with insulin sensitivity, and a loss-of-function genetic polymorphism in EPHX2 is associated with insulin sensitivity^30^. Published review clearly clarified the effects of EETs and the potential therapeutic use on diabetic and ischemic cardiomyopathy^33^. Our results showed that EETs significantly attenuated HFD-induced metabolic dysfunction, insulin resistance and inflammation, while its mechanisms need to be further explored. In our research, EETs decreased the subcutaneous and visceral fat mass in HFD mice while have no difference in food intake. Real-time PCR experiment showed EETs restored HFD-induced decrease in expression of UCP-1, UCP-2 and PGC-1α in adipose tissue, which indicated EETs decreased the body weight partially via increasing energy utilization. Also, EETs restored HFD-mediated adipogenesis with lowed genes expression of PPAR-γ, aP2 and MEST as previous study^10^. The gold standard for testing energy metabolism is using metabolic cage to calculate the thermogenesis, activity of the mice, oxygen consumption/amount of exhaled carbon dioxide, food and water, excrement and urine, all taken into assess the body’s energy metabolism. These indicators need to further improve in the further study. Adipose tissue is an important endocrine organ besides its role in storing energy, HFD-induced obesity primed inflammation in adipose tissue prior to liver in mice^34^, suggested that the dysfunction of adipose tissue strongly contributes to the initiation and exacerbation of insulin resistance. The large epididymal fat pads of male mice are frequently sampled as representative of visceral fat in obesity research^35^. ATMs associated chronic inflammation in adipose tissue is critical in the pathogenesis of insulin resistance^25^,^26^. Pro-inflammatory M1 macrophages are central mediators of obesity-induced inflammation and insulin resistance^36^, depletion of CD11c^+^ cells results in rapid normalization of obesity-induced insulin sensitivity, paralleled by a decrease in adipose and systemic inflammation^37^. Conversely, deletion of genes encoding transcription factors such as PPARγ that promote the alternative activation of M2 macrophages predisposes lean mice to the development of glucose intolerance and insulin resistance^38^. Hence, the imbalance of M1/M2 macrophages is critical for metabolic dysfunction upon the development of obesity. Promoting the resolution of adipose inflammation is therefore a potential therapeutic approach that could alleviate obesity-associated organ dysfunction. Here we showed that EETs significantly shift the macrophage phenotype from M1 (CD11c^+^) to M2 (CD206^+^) in HFD induced obesity with decreased macrophage numbers. The EETs induced shift in WAT macrophage phenotype correlated with other attributes of resolution, such as attenuation of the inflammation in adipose tissue and systemic levels. Results also showed a shift from CD3^+^CD4^−^CD8^+^ T cells toward to CD3^+^CD4^+^CD8^−^, decreased pro-inflammatory monocytes and neutrophils in circulation with EETs treatment. However, it is important to note that we were unable to include intracellular markers in this flow cytometry panel, which prevented T cell subset characterization. Our results support the ideas that the changes in immune system includes the accumulation of Th1-polarized CD4^+^ T cells, neutrophils, loss of regulatory T cells and so on, might precede the event of macrophage polarization, and ATM might be effectors of a coordinated inflammatory response^39^. These adaptive immune responses might be beneficial, at least initially, and function to preserve metabolic homeostasis^39^.

It is important to recognize that in addition to their well-established actions on leukocytes and cardiomyocytes, EETs affect numerous cell types, including adipocytes^40^. It is therefore reasonable to ask whether EETs-mediated attenuation of WAT inflammation occurred through modulation of the macrophage phenotype and/or via direct manipulation of the adipocyte cell function. Oversized adipocytes release FFAs by failing to store them. The excessive amount of FFAs targets macrophage signaling by promoting their pro-inflammatory activation, and a vicious circle evolves between hypertrophied adipocytes and adipose tissue macrophages^17^. In this study, we used a similar experimental setup, composed of murine BMDM and adipocytes, in order to better mimic WAT in mice. Interestingly, the indirect co-culture system using CM from adipocytes significantly enhanced macrophage chemotaxis, 11,12-EET or TUPS significantly inhibited macrophages migration, as well as preserved the insulin sensitivity of adipocytes impaired by conditioned medium from macrophages. *In vitro* experiment also showed that EETs inhibited M1 macrophage activation induced by LPS, while preserved IL-4 induced M2 macrophage polarization. Altogether, these data indicated the existence of a crosstalk between macrophages and adipocytes to modulate immune and metabolic function.

The signals controlling resolution of inflammation in the adipose tissue remain poorly defined, but are likely to involve reduced recruitment, local macrophage death and proliferation, as well as egress of macrophages from the inflammatory site^4^. To date, MCP-1-CCR2 system is the most well studied chemokine-chemokine receptor system in ATM recruitment. Overexpression of MCP-1 in AT causes macrophages recruitment and IR in aP2-MCP-1 mice^41^, while the decrease of MCP-1 attenuates inflammation by suppressing macrophages recruitment to AT^42^. In addition, CCR2 is a functional receptor shared by several other chemokines including MCP-3, CCL7 and CCL8, which are all expressed in obese AT and may affect macrophages recruitment^18^. In addition to MCP-1-CCR2 system, the compelling evidence indicates that RANTES-CCR5 system also plays a crucial role in obesity-induced AT inflammation and IR by regulating both macrophages recruitment and phenotype. RANTES participates in the recruitment of blood monocytes through triggering adhesion and transmigration of blood monocytes to/through endothelial cells of human WAT^43^,^44^. Notably, EETs exhibited decreased accumulation of macrophages in adipose tissue compared with HFD and down-regulated genes expression of CCL2, CCL7, CCL8 and CCL3, as well as the chemokine receptors CCR2 and CCR5 in WAT. We also showed that the high prevalence of CLS were highly correlated to AT inflammation and metabolic disorder and considered to be pathological lesions in AT of obese mice. Adipose tissue inflammation has been proposed to involve with proliferation of local macrophages^23^. Interestingly, CYP2J2/EETs significantly attenuated HFD induced macrophage proliferation, specifically in the region of CLS. This crosstalk between adipocytes and macrophages establishes and maintains the chronic inflammation state in obese AT through persistently recruiting new macrophages/monocytes from circulation and local proliferation, EETs play important roles in maintain adipose tissue homeostasis.

Our study demonstrated that EETs regulated adipose tissue macrophages polarization and maintained metabolic homeostasis, but the specific mechanism need to be further elucidated. We performed microarray *in vitro* experiment with BMDM, and found that cAMP-EPAC signaling may be associated with the effects of EETs on FFA-induced macrophages polarization, which indicated its role in metabolic homeostasis. The pleiotropic second-messenger cAMP plays a crucial role in relaying the extracellular cues to trigger various intracellular signaling. The major intracellular functions of cAMP are transduced by protein kinase A (PKA) and by exchange proteins directly activated by cAMP (EPAC)^45^. Depending upon cellular context and their relative abundance, distribution, and localization, these two intracellular receptors may act independently, synergistically, or antagonistically in regulating a specific cellular function^45^. cAMP-EPAC act as an important stress response signaling involved in many kinds of pathological processes, such as energy metabolism, cardiac stress, chronic pain, cancer and infections^46^. In line with our findings, Yan *et al*. showed that EPAC1 null mice were resistant to HFD-induced obesity, hyperleptinemia and glucose intolerance, uncovered an important EPAC1 function in energy homeostasis and leptin resistance^47^.

Collectively, our results suggest that CYP2J2-EETs-sEH metabolic pathway maintains metabolic and immune homeostasis via regulating adipose tissue macrophages polarization, and ultimately diminishes inflammation and the associated insulin resistance, which are associated with the inhibition of cAMP-EPAC signaling pathway. With the content of better understanding the mechanism of CYP2J2-EETs-sEH metabolic pathway on obesity-induced insulin resistance will provide a therapeutic strategy in combating obesity related metabolic diseases.

## Additional Information

**How to cite this article**: Dai, M. *et al*. CYP2J2 and Its Metabolites EETs Attenuate Insulin Resistance via Regulating Macrophage Polarization in Adipose Tissue. *Sci. Rep.* **7**, 46743; doi: 10.1038/srep46743 (2017).

**Publisher's note:** Springer Nature remains neutral with regard to jurisdictional claims in published maps and institutional affiliations.

## Supplementary Material

Supplementary Data
