# Supplementary material for: CYP2J2 and Its Metabolites EETs Attenuate Insulin Resistance via Regulating Macrophage Polarization in Adipose Tissue
Source: Sci Rep. 2017 Apr 25;7:46743. doi: 10.1038/srep46743 (PMC5404269; doi:10.1038/srep46743)
Supplement: Supplementary Data [file srep46743-s1.doc]

**Supplementary information**

**CYP2J2 and its metabolites EETs attenuate insulin resistance via regulation macrophage polarization in adipose tissue**

Meiyan Dai^1^, Lujin Wu^1^, Peihua Wang^1^, Zheng Wen^1^, Xizhen Xu^1^ and Dao Wen Wang^1^

Supplemental Table 1. Primer sequences for quantitative real-time RT-PCR.

| Gene | Forward | Reverse |
| --- | --- | --- |
| CCR2 | TGCCATCATAAAGGAGCCA | AGCACATGTGGTGAATCCAA |
| MCP1 | GTGCTGACCCCAAGAAGGAA | GTGCTGAAGACCTTAGGGCA |
| CCL7 | AAGTGGGTCGAGGAGGCTAT | CCATTCCTTAGGCGTGACCA |
| CCL8 | CTGAAGATCCCCCTTCGGGT | CCCACTTCTGTGTGGGGTC |
| CCR5 | GTTGTTTTGGAGAACGCCCC | CAACACTGCTCCGAAACTGC |
| CCL3 | GTAGCCACATCGAGGGACTC | GATGGGGGTTGAGGAACGTG |
| CCL4 | GCAACACCATGAAGCTCTGC | CCATTGGTGCTGAGAACCCT |
| CCL5 | GCAGTCGTGTTTGTCACTCG | CCGAGTGGGAGTAGGGGATT |
| CD68 | ATCTTGCTAGGACCGCTTAT | CTGGCTGTGCTTTCTGTG |
| F4/80  IL-6  Nos2  CD11c  IL-1β  TNFα  IL-10  Ym-1  MRC2  MRC1  MGL1  MGL2 | CTTTGGCTATGGGCTTCCAGTC  GTTGCCTTCTTGGGACTGATG  CCAAGCCCTCACCTACTTCC  AGATGTTGGAGGAAGCAAATGG  TTTGAAGTTGACGGACCCCA  GTCTACTGAACTTCGGGGTGAT  GCTCTTACTGACTGGCATGAG  AGAAGCTCTCCAGAAGCAATCC  TACAGCTCCACGCTATGGATT  GTGGAGTGATGGAACCCCAG  TGAGAAAGGCTTTAAGAACTGGG  TTAGCCAATGTGCTTAGCTGG | GCAAGGAGGACAGAGTTTATCGTG  CAACTCTTTTCTCATTTCCACGA  CTCTGAGGGCTGACACAAGG  TGGGCGGGTTCAAAGACG  TGTGCTGCTGCGAGATTTGA  ACTTGGTGGTTTGTGAGTGTGA  CGCAGCTCTAGGAGCATGTG  CTGTCCTTAGCCCAACTGGT  CACTCTCCCAGTTGAGGTACT  CTGTCCGCCCAGTATCCATC  GACCACCTGTAGTGATGTGGG  GGCCTCCAATTCTTGAAACCT |
| AGR1  UCP-1  UCP-2  PGC-1α  aP2  MEST  PPARγ  GAPGH | CTCCAAGCCAAAGTCCTTAGAG  TGTGGCTTCTTTTCTGCGA  ATGGTTGGTTCAAGGCCAC  GCCCGAGCAATCTGAGTTGTAC  GAAATCACCGCAGACGACA  CCATCCTCACCAGGCTCAT  TCTTCCATCACGGAGAGGTC  GTGTTTCCTCGTCCCGTAGA | AGGAGCTGTCATTAGGGACATC  TTTGATTTCTTTGGTTGGTTTTA  TCATGAGGTTGGCTTTCAGG  GGCCGTTTAGTCTTCCTTTCCT  GCCTCTTCCTTTGGCTCAT  ACCAGGTTGCCGTCATTGT  GATGCACTGCCTATGAGCAC  TTCCCATTCTCGGCCTTGAC |

**
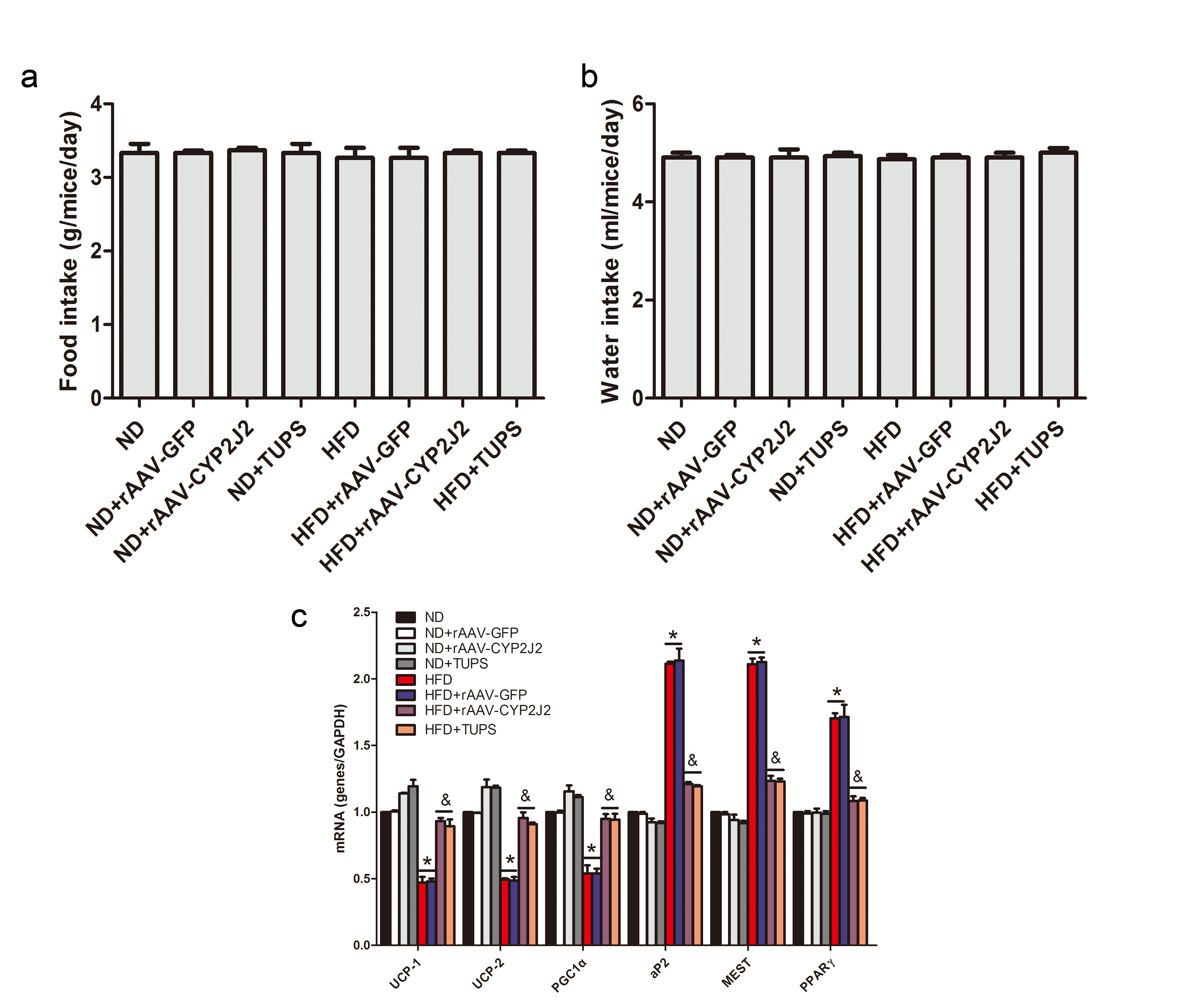
**

**Supplemental Figure 1. Food consumption and energy metabolism in mice.** RAAV-CYP2J2 overexpression or TUPS administration had no effect on mice food (a) and water intake (b). Genes expression of energy metabolism and adiogenesis in adipose tissue of mice (c). (n=15 for each group, **p<*0.05 *vs* ND; &*p<*0.05 *vs* HFD)

**
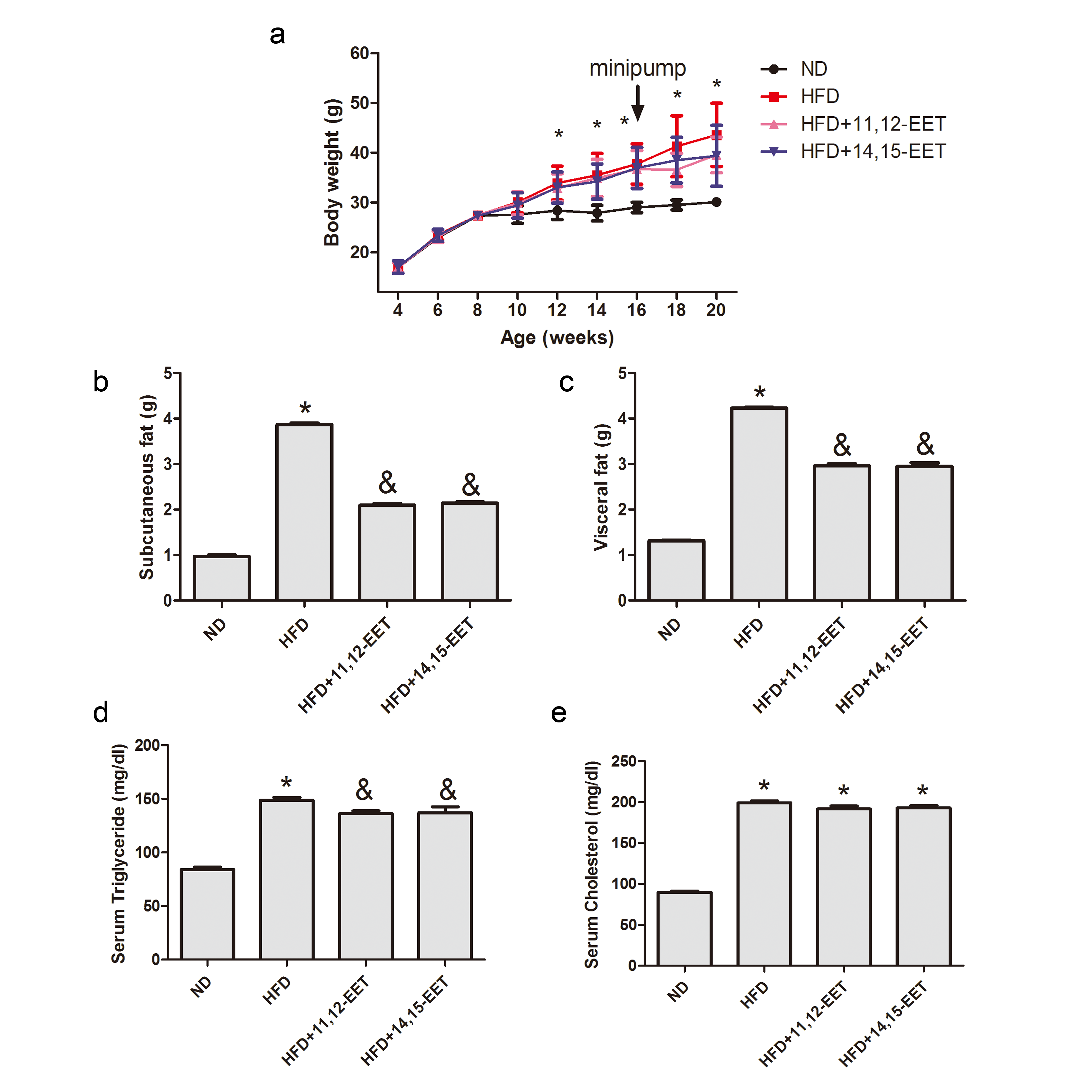
**

**Supplemental Figure 2. EETs infusion with mini-pump attenuated metabolic dysfunction in HFD mice.** (a) The curves of body weight gain over time. The subcutaneous (b) and visceral (c) fat content of mice under various treatment conditions. The concentration of serum triglyceride (d) and cholesterol (e) in mice. (n=15 for each group; **p<*0.05 *vs* ND; &*p<*0.05 *vs* HFD)

**
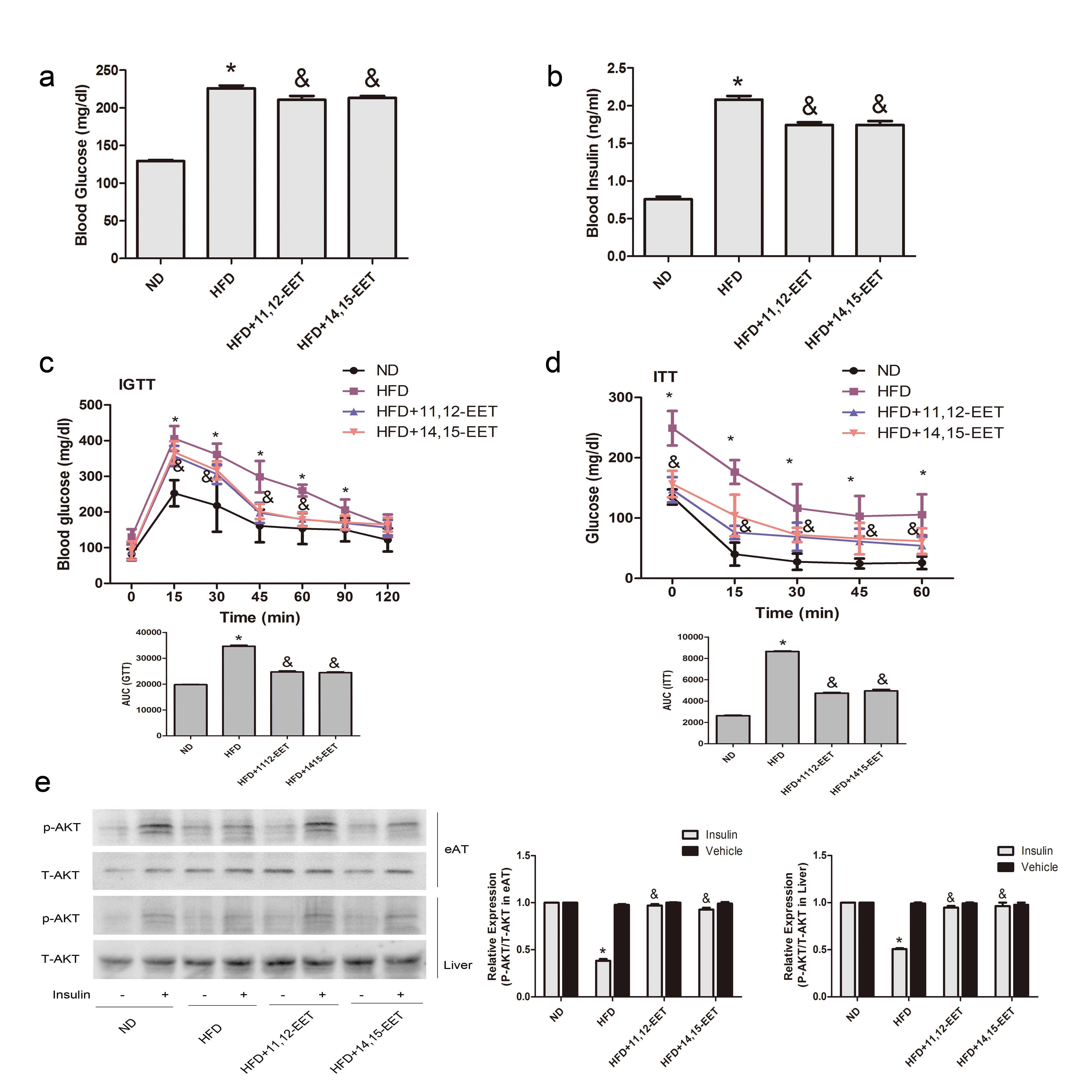
**

**Supplemental Figure 3. EETs infusion with mini-pump attenuated HFD-induced insulin resistance.** Plasma concentration of glucose (a) and insulin (b) in mice after 16 weeks of ND or HFD. (c) GTT and ITT (d) assay were performed in mice fed ND or HFD for 16 weeks. (e) Representative immunoblots and quantitation for Akt phosphorylation level in mice epididymal adipose tissue and liver. (n=15 for each group; **p<*0.05 *vs* ND; &*p<*0.05 *vs* HFD)

**
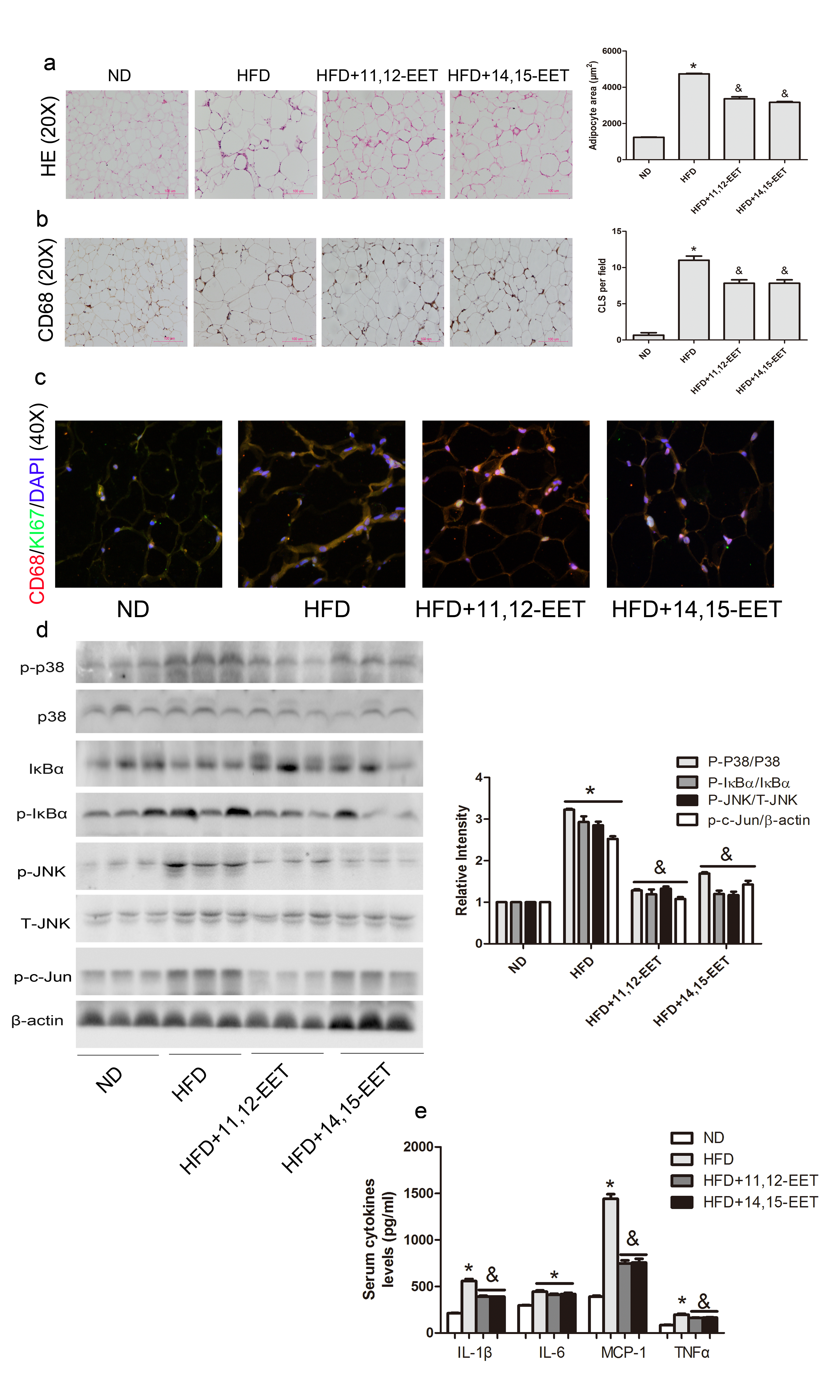
**

**Supplemental Figure 4. EETs infusion with mini-pump attenuated HFD-induced adipose tissue and systemic inflammation.** (a) Representative images of HE staining in adipose tissue and the calculated average adipocyte area. (b) Representative CD68 immunohistochemical staining and the numbers of CLS in adipose tissue with indicated interventions. (c) Representative images of macrophage proliferation staining with CD68 and Ki67 in adipose tissue. (d) Representative immunoblots and quantitation for MAPK and NF-κB signaling pathways in adipose tissue. (e) ELISA analysis of inflammatory cytokines in serum. (n=15 for each group; **p<*0.05 *vs* ND; &*p<*0.05 *vs* HFD)
